# Comparative Clinical Evaluation of Chamomile, Sage, and Ginger Mouthwashes in Reducing Plaque and Gingival Inflammation

**DOI:** 10.3390/antibiotics15050433

**Published:** 2026-04-27

**Authors:** Ioana Elena Lile, Anda Olivia Jesamine Samoilă, Carolina Cojocariu, Gianina Tapalagă, Neli-Kinga Oláh, Otilia Lavinia Stana, Adelina Berari, Diana Marian

**Affiliations:** 1 Department of Dentistry, Faculty of Dentistry, “Vasile Goldiș” Western University of Arad, 94-96 Revolutiei Blvd., 310025 Arad, Romania; lile.ioana@uvvg.ro (I.E.L.); stana.otilia@uvvg.ro (O.L.S.); berari.adelina@uvvg.ro (A.B.); marian.diana@uvvg.ro (D.M.); 2 Multidisciplinary Doctoral School, Vasile Goldis Western University of Arad, 310414 Arad, Romania; samoila.anda@uvvg.ro; 3 Department of Odontotherapy and Endodontics, Faculty of Dental Medicine, “Victor Babes” University of Medicine and Pharmacy Timisoara, Eftimie Murgu Square 2, 300041 Timisoara, Romania; 4 Department of Pharmaceutical Chemistry, Faculty of Pharmacy, Vasile Goldiș Western University of Arad, L. Rebreanu Str. 86, 310414 Arad, Romania; olah.neli@uvvg.ro

**Keywords:** chamomile, sage, ginger, dental plaque, gingival inflammation, natural mouthwash, oral hygiene, Silness–Löe plaque index, gingival Index, biocompatibility

## Abstract

Background: Despite mechanical hygiene, plaque-related illnesses like gingivitis and periodontitis affect over 3.5 billion people globally. Natural mouthwashes are becoming increasingly popular as consumers shift toward plant-based alternatives to chlorhexidine, which may have drawbacks that limit long-term acceptability. This study aimed to evaluate the short-term clinical potential of three herbal mouthwashes—*Matricaria chamomilla* (chamomile), Salvia officinalis (sage), and *Zingiber officinale* (ginger)—in reducing dental plaque and clinical signs of gingival inflammation in young adults. (2) Materials and Methods. A randomised controlled clinical trial was conducted on 175 systemically healthy participants, allocated equally into five groups (three herbal groups, placebo, and chlorhexidine). Each herbal group used a 2% aqueous infusion three times daily for twelve weeks. The 2% aqueous infusion concentration was selected based on commonly reported concentrations in previous phytotherapeutic and clinical studies evaluating herbal mouthwashes, balancing potential efficacy with safety and tolerability. The plant materials were sourced from certified suppliers, and standardized dried plant parts were used under controlled preparation conditions. Clinical assessments were performed at baseline (T0), week 1 (T1), week 5 (T2), and week 9 (T3), corresponding to the beginning of each evaluation interval within the 12-week study, using the Silness–Löe Plaque Index and the modified Löe–Silness Gingival Index. Data were analyzed using repeated-measures ANOVA with Bonferroni post hoc correction. (3) Results. Repeated-measures ANOVA revealed a significant main effect of time for both plaque accumulation and gingival index scores. For the Silness–Löe Plaque Index, a marked time-dependent reduction was observed across the active treatment groups (*p* < 0.001; η^2^_p_ = 0.56), with a significant time × group interaction (*p* < 0.001; η^2^_p_ = 0.49). Similarly, the modified Löe–Silness Gingival Index showed a significant reduction over time (*p* < 0.001; η^2^_p_ = 0.22), with a significant interaction effect between time and mouthwash type (*p* < 0.001; η^2^_p_ = 0.17). No statistically significant differences were found among the three herbal mouthwashes in post hoc Bonferroni comparisons (all *p* > 0.05), whereas all active treatments showed significantly better outcomes compared with the placebo. (4) Discussion. All three rinses showed similar clinical effects on plaque and gingival scores. However, without mechanistic assays, no claims can be made about comparable antibacterial or anti-inflammatory activity. Compared with conventional antiseptics such as chlorhexidine, herbal rinses offer important advantages in terms of biocompatibility, safety, and tolerability, with no staining, taste alteration, or mucosal irritation reported. At T3, the correlation between plaque and gingival indices was weak (Spearman’s ρ = 0.18, *p* = 0.09), suggesting limited linear association; this finding should be interpreted cautiously, as the low end-range values and limited variability of both indices at this time point may have masked a true association. This exploratory observation raises, but does not confirm, the possibility that factors other than plaque reduction may contribute to gingival improvement. (5) Conclusions. Significant reductions in dental plaque and clinical signs of gingival inflammation were observed following regular use of chamomile, sage, and ginger mouthwashes for twelve weeks. All herbal formulations exhibit similar clinical results. Longer-term controlled trials incorporating microbiological and phytochemical analyses are recommended to validate these findings further.

## 1. Introduction

In 2021, the World Health Assembly adopted a landmark resolution calling for the integration of oral health into the non-communicable disease (NCD) agenda and universal health coverage. It mandated the WHO to translate the Global Strategy on Oral Health (A75/10 Add 1) into an actionable plan with quantifiable targets for 2030 [1]. Using information from the GBD project, IARC, and WHO surveys, the WHO Global Oral Health Status Report (GOHSR) that followed offered the first thorough worldwide evaluation of the burden of oral illness and is currently a strategic tool for public health stakeholders and policymakers [1].

Oral diseases affect 3.5 to 3.7 billion individuals globally, with over a billion having severe periodontal disease [2]. Despite preventive measures, plaque-related disorders including gingivitis and periodontitis remain highly prevalent.

Mechanical plaque control remains the cornerstone of oral hygiene, particularly through toothbrushing and interdental cleaning methods such as dental floss and interdental brushes. Mouthwashes may serve as adjuncts rather than substitutes, with limited penetration into subgingival areas. However, mechanical plaque removal alone is often insufficient to adequately prevent or control periodontal disease [3]. Supplemental chemotherapeutic products, such as fluoride and chlorhexidine mouthwashes, may reduce bacterial burden but their long-term effects are unknown, and chlorhexidine causes discolouration and taste alterations [3,4]. Due to these restrictions, herbal mouthwashes with less adverse effects and antibacterial properties are becoming popular [5,6]. Herbal rinses contain antibacterial, antioxidant, and clinically relevant bioactive compounds, including essential oils, polyphenols, and flavonoids. Extracts such as chamomile, sage, and clove have been reported to inhibit oral pathogens, including Porphyromonas gingivalis, Fusobacterium nucleatum, and Streptococcus mutans, while also modulating local tissue responses [6].

Plant-based formulations are effective, according to recent clinical trials [7,8,9,10,11]. Additionally, a 2025 clinical investigation found that spilanthol and cannabidiol mouthwash improved gingival health without adverse effects [7].

Given the established associations between chronic oral inflammation and systemic diseases such as diabetes, cardiovascular disease, poor pregnancy outcomes, and neurological disorders, safe and effective plaque reduction supplements are important [12,13,14,15].

Polyherbal mouthwashes reduced full-mouth bleeding and plaque scores (FMBS, FMPS) in moderate-to-severe periodontitis patients over 3 months, demonstrating their efficacy as mechanical-cleaning adjuncts [16]. Despite its importance, mechanical plaque removal is rarely sufficient to prevent or treat periodontitis [16,17].

To our knowledge, few studies have directly compared multiple individual herbal formulations within the same randomized controlled clinical design. In this context, the present study compares three herbal mouthwashes based on *Matricaria chamomilla* (chamomile), Salvia officinalis (sage), and *Zingiber officinale* (ginger) in their ability to reduce dental plaque and improve gingival clinical parameters in young adults. Standardized clinical measurements included the Silness–Löe Plaque Index and the modified Löe–Silness Gingival Index (Lobene modification).

## 2. Results

### 2.1. Phytochemical Profile of the Investigated Herbal Extracts

The results derived from the spectrophotometric and LC–MS/MS analyses are presented in detail in Table 1, summarizing the concentrations of the identified phenolic and flavonoid compounds.

The Chamomile and Sage aqueous extracts are rich in polyphenols, phenolic acids and flavonoids. As was expected the Ginger extract is poor in these compounds.

A trace of trans-para-coumaric acid was observed in all extracts. The Chamomile extract also contains trace amounts of caffeic acid and rutoside, and the Sage extract contains rutoside, quercetin-3-glucoside, and hyperoside. The content of the other identified polyphenols is shown in Table 2.

In Chamomile and Sage extracts, a wide range of individual polyphenols were identified, belonging to phenolic acids (caffeic acid, chlorogenic acid and its isomers, ferulic acid or rosmarinic acid), flavonoids (acacetin, apigenin, diosmin, hyperoside, luteolin and quercetin derivatives, tilianin), or isoflavones such genistin. Some compounds are specific to the species studied, such as apigenin in Chamomile and rosmarinic acid and luteolin derivatives in Sage.

### 2.2. Participant Characteristics

The participants were divided equally among the five study groups: three groups using herbal mouthwashes—*Matricaria chamomilla* (chamomile), *Salvia officinalis* (sage), and *Zingiber officinale* (ginger)—as well as a placebo group and a chlorhexidine control group, with n = 35 participants in each group. The majority of participants were from urban areas, and gender distribution was relatively balanced across groups. Most individuals reported brushing their teeth twice daily and using mouthwash at least once or twice per day.

No statistically significant differences were observed between the groups at baseline regarding age, gender, residence, or oral hygiene habits (*p* > 0.05), confirming that the groups were comparable before the start of the intervention (Table 3).

Baseline descriptive comparisons were based on the full enrolled sample (N = 175), whereas longitudinal analyses were conducted on completers only (n = 171).

### 2.3. Baseline Plaque and Gingival Indices

**Figure 1:**
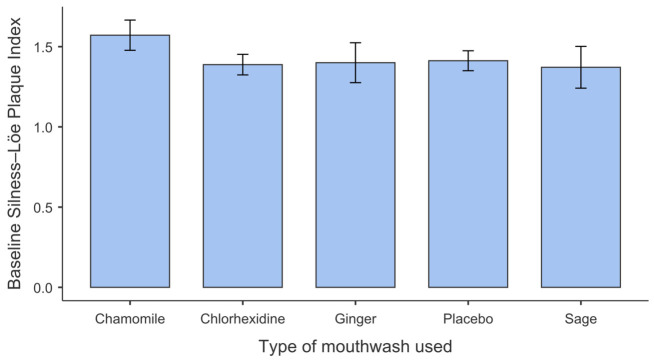
Baseline Silness–Löe Plaque Index across mouthwash groups.

**Figure 2:**
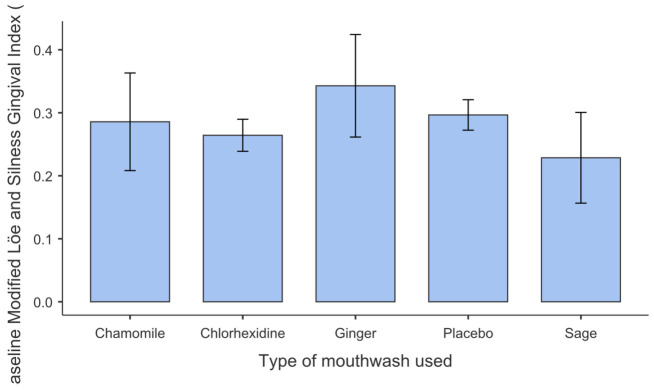
Baseline Modified Löe and Silness Gingival Index (Lobene).

At baseline, the mean Silness–Löe Plaque Index values were 1.57 ± 0.56 for the chamomile group, 1.37 ± 0.77 for the sage group, 1.40 ± 0.74 for the ginger group, 1.41 ± 0.37 for the placebo group, and 1.39 ± 0.38 for the chlorhexidine group (Table 4). For the modified Löe and Silness Gingival Index (Lobene), the mean baseline scores were 0.29 ± 0.46, 0.23 ± 0.43, 0.34 ± 0.48, 0.30 ± 0.14, and 0.26 ± 0.15, respectively, across the five study groups (Table 4). Baseline plaque scores varied slightly between groups; however, these differences did not reach statistical significance (*p* > 0.05), as illustrated in Figure 1. Similarly, baseline gingival inflammation showed low and comparable values across all five groups (*p* > 0.05), as shown in Figure 2. The Shapiro–Wilk test indicated deviations from normality for both indices at baseline (*p* < 0.001). Given the balanced group sizes and sample size per group (n = 35), repeated-measures ANOVA was considered robust to moderate deviations from normality according to the central limit theorem. Levene’s test revealed unequal variances for the plaque index (*p* < 0.05), but not for the gingival index (*p* > 0.05); therefore, Welch’s ANOVA was applied where appropriate.

No statistically significant differences were observed among the five groups at baseline for either the plaque index or the gingival index (all *p* > 0.05), confirming clinical comparability at the start of the study.

### 2.4. Changes in Plaque Index over Time

Repeated measures ANOVA (Table 5) revealed a statistically significant main effect of time on the Silness–Löe Plaque Index, indicating a substantial change in plaque accumulation throughout the study period (Greenhouse–Geisser correction, *p* < 0.001, η^2^_p_ = 0.56). Importantly, a significant time × type of mouthwash interaction was observed (*p* < 0.001, η^2^_p_ = 0.49), demonstrating that the pattern and magnitude of plaque reduction differed markedly among the five study groups.

Of the 175 enrolled participants, 171 completed all follow-up assessments and were included in the repeated-measures ANOVA. This explains the residual degrees of freedom (df = 510) reported in Table 5 and Table 6.

**Figure 3:**
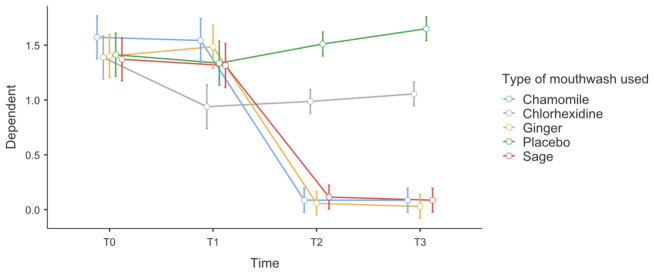
Evolution of the Silness–Löe Plaque Index over four time points (baseline, T1, T2 and T3).

Between-subjects analysis further showed a significant association of the mouthwash type on plaque levels (*p* < 0.001, η^2^_p_ = 0.47). Visual inspection of the estimated marginal means indicated that participants using chlorhexidine and herbal mouthwashes (chamomile, ginger, and sage) experienced a pronounced reduction in plaque index values, particularly between T1 and T2, followed by stabilization at low levels through T3. In contrast, the placebo group exhibited a progressive increase in plaque accumulation over time. Overall, these findings indicate that while plaque levels changed significantly over time in all groups, the effectiveness of plaque control was strongly dependent on the type of mouthwash used, as illustrated in Figure 3.

To determine whether a similar temporal pattern was evident in gingival health, a separate repeated-measures ANOVA was conducted for the Gingival Index.

### 2.5. Changes in Gingival Index over Time

Repeated measures ANOVA (Table 6) revealed a statistically significant main effect of time on the modified Löe and Silness Gingival Index (Lobene), indicating significant changes in gingival inflammation throughout the study period (Greenhouse–Geisser correction, *p* < 0.001, η^2^_p_ = 0.22). A significant time × type of mouthwash interaction was also observed (*p* < 0.001, η^2^_p_ = 0.17), demonstrating that the temporal pattern of gingival inflammation differed among the five study groups.

Between-subjects analysis showed a significant effect of mouthwash type on gingival index values (*p* < 0.001, η^2^_p_ = 0.19). Inspection of the estimated marginal means indicated that participants using chlorhexidine and herbal mouthwashes exhibited a marked reduction in gingival index scores over time, with a pronounced improvement observed around T2, followed by partial stabilization at T3. In contrast, the placebo group showed comparatively smaller improvements across the study period.

**Figure 4:**
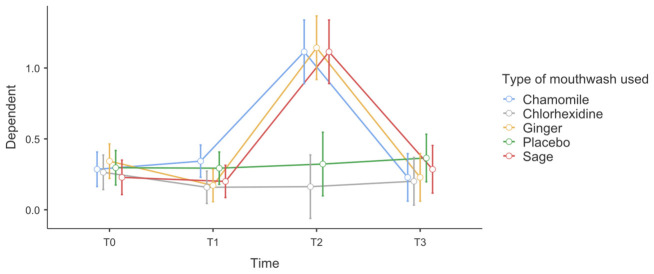
Evolution of the modified Löe and Silness Gingival Index (Lobene).

Overall, these results indicate that gingival clinical parameters were significantly influenced by both time and the type of mouthwash used. Significant reductions in gingival scores compared with placebo were associated with the active treatments, as illustrated in Figure 4.

### 2.6. Comparative Effectiveness Between Herbal Mouthwashes

To further explore potential differences among the herbal formulations, a comparative analysis was conducted including the chamomile, sage, ginger, chlorhexidine, and placebo groups. For the Silness–Löe Plaque Index, although the overall time × group interaction was statistically significant when all five groups were considered, post hoc Bonferroni comparisons restricted to the three herbal groups did not reveal statistically significant differences at corresponding time points (all *p* > 0.05). Similarly, for the modified Löe–Silness Gingival Index (Lobene), all three herbal mouthwashes showed reductions in clinical scores over time. However, the differences among the three herbal groups did not reach statistical significance after correction for multiple comparisons (all *p* > 0.05). Post hoc Bonferroni tests confirmed the absence of significant pairwise differences among the herbal groups (Table 7).

## 3. Discussion

This randomized clinical study evaluated the short-term effects of three herbal mouthwashes of chamomile, sage, and ginger on the formation of oral plaque and gingival inflammation in young adults. While significant differences were observed between active treatments and placebo, no statistically significant differences were detected among the three herbal formulations. All three formulations resulted in statistically significant time-dependent reductions in both the Silness–Löe Plaque Index and the modified Löe and Silness Gingival Index, without group differences with respect to statistical significance. The large effect size (η^2^_p_ = 0.56) indicates that more than half of the variance in plaque index reduction can be attributed to the time effect, highlighting the strong temporal improvement observed across mouthwash groups. In this study, all three herbal rinses were associated with a progressive reduction in plaque scores over time, when used in combination with participants’ routine mechanical oral hygiene. Both the sharp and sustained reduction observed in plaque load in the first week and the stabilization in T2 and T3 are consistent with previous investigations observing that herbal or polyherbal preparations have short-term biofilm break effects followed by a plaque accumulation plateau as biofilm matures [5,6,15]. The age range (20–45 years) was selected to ensure a relatively homogeneous population with stable oral hygiene habits and minimal confounding from age-related periodontal changes.

Studies comparing herbal treatment with conventional drug therapy have produced parallel responses that show that in the short term, reductions in plaque mimic those of chemical extracts but with no deleterious effects [6,7,8,18]. The observed effects are biologically plausible in light of the known phytochemical composition of these plant extracts. Essential oils, polyphenols, flavonoids, and terpenoids are all common components for herbal formulations. These compounds perform antibacterial, antioxidant, and anti-inflammatory effects in oral biofilm production [9,10,11,19,20]. These compounds have been shown to perturb microbial membranes, hinder the synthesis of extracellular polymeric compounds, inhibit quorum sensing, and perturb host inflammatory systems. Chamomile (*Matricaria chamomilla*) contains α-bisabolol, chamazulene, apigenin, luteolin, and quercetin. The compounds help stop pro-inflammatory mediators like IL-1β and TNF-α, promoting tissue formation in the soft tissue recovery process [21,22,23]. Systematic evaluations show that chamomile has complementary advantages for the treatment of gingivitis and early periodontitis, acting often like chlorhexidine for transient inflammatory control but with fewer adverse effects [23].

Sage (*Salvia officinalis*) contains various volatile and non-volatile compounds that act as antibacterial and anti-inflammatory agents, such as rosmarinic acid, cineole, and thujone. Clinical investigations of S. officinalis mouthwash compared with chlorhexidine have indicated reductions in plaque and gingival bleeding [24,25]. However, they have not consistently shown superiority over traditional agents. These findings endorse sage as a safe adjunct for short-term gingival irritation management. Ginger (*Zingiber officinale*) is rich in gingerols, shogaols, paradols, zingerone, and zerumbone. The chemicals are found to inhibit bacteria-causing periodontal disease and inflammation through the NF-κB signaling pathways, as well as in prostaglandin [26,27,28,29]. The observed clinical improvements may be partially explained by the presence of bioactive compounds such as apigenin, rosmarinic acid, and gingerol derivatives, which have been shown to modulate inflammatory pathways, inhibit microbial growth, and interfere with biofilm formation.

Evidence from reviews and experimental trials shows the potential for periodontal inflammation, oxidative stress, and gingival hemorrhage to be attenuated by ginger-based formulations; however, results vary from formulation to formulation and from population to population [27]. In clinical settings, herbal rinses significantly outperform an antiseptic like chlorhexidine in terms of tolerability. Chlorhexidine remains the optimal drug for control of plaque, although it cannot be used for extended periods of time due to possible tooth discoloration, taste changes, mucosal irritation, and dysbiosis [5,6]. Although chlorhexidine demonstrated the highest overall efficacy, the herbal formulations showed comparable trends in reducing plaque and gingival inflammation, while offering a superior tolerability profile, supporting their potential role as long-term adjuncts in oral hygiene. Alternatively, this research found no adverse reactions reported, confirming findings from earlier studies that showed a lower impact of natural formulations on soft tissues and the oral microbiome [8,20,30]. The absence of significant intergroup variances in this study likely reflects similar phytochemical properties across chamomile, sage, and ginger, including essential oils and polyphenols, which evoke similar antibacterial and anti-inflammatory action. Further clinical and mechanistic studies targeting these plants separately have reported similar improvements in the plaque and gingival bleeding indices [21,22,23,24,28].

These results further support the growing body of evidence emphasizing the role of plant-based mouthrinses as viable adjuncts to mechanical plaque control. Recent clinical investigations have confirmed that natural mouthrinses provide comparable efficacy to chlorhexidine in reducing plaque accumulation and gingival inflammation, while maintaining a superior biocompatibility profile [31,32].

One noteworthy finding of this study was the absence of a statistically significant association between plaque reduction and gingival improvement at the final 12-week visit. From a statistical perspective, this finding reflects the absence of a linear correlation rather than evidence of a true biological dissociation between the two processes. At T3, both plaque and gingival indices were clustered at low values, likely generating a floor effect that reduced score dispersion and may have masked potential associations.

The stabilization of clinical indices observed at T3 may suggest a plateau in therapeutic response, possibly reflecting the limitations of aqueous formulations or adaptive changes within the oral microbiome. However, this interpretation remains speculative and requires confirmation through future studies incorporating microbiological assessments and host-response biomarkers.

In addition, plaque accumulation and gingival inflammation, although closely related, represent partially distinct clinical constructs influenced not only by biofilm burden but also by host immune response, microvascular changes, and oxidative–antioxidant balance. Given the exploratory nature of this analysis and the absence of microbiological and biomarker data, no firm mechanistic inference, including a direct anti-inflammatory effect of the herbal rinses, can be drawn from the correlation findings alone. Taken together, these considerations suggest that the observed improvements in gingival health cannot be fully explained by plaque scores alone and should be interpreted within a multifactorial framework, rather than as evidence of a specific mechanism of action.

Phytotherapeutic treatments, following host-modulating medicine applications [20], have reported a similar partial dissociation between plaque indices and inflammatory responses. And these findings have key clinical implications. Chamomile-, sage-, and ginger-based mouthwashes can be added to standard oral hygiene routines for those who prefer natural remedies in place of topical antimicrobials and for patients who have mucosal sensitivity and moderate-grade gingivitis who require supportive treatment. Considering the documented associations between chronic periodontal inflammation and inflammatory systemic conditions like CVD, diabetes, and poor pregnancy outcome [12,13,14,15], the pursuit of safe biocompatible adjuncts to modify gingival inflammation is still critical.

Although all active mouthrinses were associated with improvements in plaque scores and reductions in modified Löe–Silness gingival index values, these findings should be interpreted as improvements in the clinical signs of plaque-induced gingival changes rather than as direct evidence of anti-inflammatory activity. Since bleeding on probing (BOP) and biochemical inflammatory markers were not assessed, no definitive conclusions can be drawn regarding true anti-inflammatory efficacy. Therefore, the observed changes primarily reflect improvements in gingival clinical status associated with reduced biofilm accumulation. These findings contribute to the ongoing discussion on integrating natural bioactive compounds into routine dental care, in line with emerging patterns of material and product selection reported in Romanian clinical practice [33].

### 3.1. Clinical Implications

Our results hint as to how chamomile-, sage-, and ginger-based mouthwashes may work as a safe and effective adjunct to daily oral hygiene, primarily for individuals seeking natural alternatives or those who do not tolerate conventional antiseptics such as chlorhexidine. Because of their favorable safety profile and lack of side effects, as well as proven short-term reduction in both plaque accumulation and gingival inflammation, herbal rinses might offer a useful adjunct in preventive care and also for treatment of mild gingivitis. Based on the known links of chronic oral inflammation and systemic disorders (e.g., cardiovascular disease, diabetes mellitus, pregnancy complications, and neurodegenerative disorders), biocompatible anti-inflammatory adjuncts warrant further consideration in broader preventive health strategies.

### 3.2. Study Limitations

There are limitations that need to be recognized in the interpretation of these findings. First, the intervention time for this study was relatively short (twelve weeks), which limits conclusions regarding long-term stability, relapse, or cumulative effect. Second, microbiological or immunological assessments were not conducted, which would clearly have answered the question of how the herbal formulations affect biofilm composition or inflammatory biomarkers. Third, participants were young, systemically healthy adults whose generalizability is limited to older adult populations, patients with medical compromise, or patients with moderate to severe periodontal disease. Last, follow-up to rinsing/brushing instructions was self-reported, with potential reporting bias. Crucially, given the design of the trial, the benefits seen are only seen statistically and are associations, which prevents any causal inferences from being made about the direct effect of the herbal mouthwashes. Furthermore, the correlation analysis between plaque and gingival indices was exploratory and performed on end-range scores with limited variability at the final visit, which may have led to underestimation of true associations (floor effect). Although the clinical outcomes suggest a beneficial effect, the lack of inflammatory and oxidative stress marker assessment limits direct mechanistic inference. Future studies should integrate host-response biomarkers, including inflammatory cytokines and oxidative stress parameters, to better elucidate the underlying biological pathways. Also, a detailed dose–response analysis of individual bioactive compounds within the final formulations was not performed and should be addressed in future studies.

Nevertheless, despite the above limitations, this study contributes important information that supports the near-term effectiveness and safety of chamomile, sage, and ginger mouthwashes for plaque formation and gingival inflammation. Future multicenter studies with longer follow-up, systematic phytochemical characterization, and microbiological and immunological evaluations are required to clarify which association-based mechanisms are involved in the therapeutic effects [34].

### 3.3. Future Directions/Future Perspectives

Longer-term randomized controlled trials conducted on bigger and more varied populations with more patients, including elderly and systemic patients, are recommended for further clinical research. Standardized phytochemical profiling of herbal formulations is crucial for reproducibility and to establish dose–response correlations. Microbiological and immunological analyses should also be conducted to elucidate the mechanisms that might associate herbal rinses with plaque, gingival inflammation, and host responses. Furthermore, investment should also be made in studying the potential for herbal formulations for peri-implant maintenance, periodontal maintenance programs, and personalized preventive strategies. Possibly, combining herbal therapies and synergistic phytochemical approaches are also promising future applications [29,31].

## 4. Materials and Methods

This study complied with the ethical principles for research involving human participants and received approval from the Institutional Ethics Committee of “Vasile Goldiș” Western University of Arad (Approval No. 27/October 2025). Participant recruitment was conducted immediately thereafter, between October and November 2025, followed by a 12-week follow-up period completed in January 2026. Data analysis and manuscript preparation were performed subsequently. All participants were informed about the purpose, methodology, potential benefits, and risks of the research, and provided written informed consent prior to inclusion. The study adhered to the ethical principles of the Declaration of Helsinki (2013), ensuring respect for persons, beneficence, and the confidentiality of personal data. Participants were informed that they could withdraw at any time without any consequences.

### 4.1. Study Design and Objectives

This clinical, randomized, controlled study aimed to investigate the short-term effects of three herbal mouthwashes—prepared from *Matricaria chamomilla* (chamomile), Salvia officinalis (sage), and *Zingiber officinale* (ginger)—in reducing dental plaque and gingival inflammation among young adults. The study follows a twelve-week protocol with systematic rinse intervals and repeated clinical evaluations at baseline (T0), week 1 (T1), week 5 (T2), and week 9 (T3). The study has been registered in the ISRCTN registry under the number ISRCTN10078236.

In order to contextualize the clinical performance of the herbal formulations, two additional control groups were included, a placebo mouthwash and a chlorhexidine-based mouthwash, serving as negative and positive controls, respectively. Randomization was performed using a computer-generated allocation sequence. The randomization list was created by an independent investigator not involved in participant recruitment, clinical evaluation, or statistical analysis.

Clinical evaluation was performed using the Silness–Löe Plaque Index and the modified Löe and Silness Gingival Index (Lobene) as standardized outcome measures. Only individuals presenting plaque-induced gingival changes without clinical attachment loss or radiographic evidence of periodontitis were included. Periodontal status was assessed using the modified Löe–Silness Gingival Index and Plaque Index, in accordance with the 2018 classification of periodontal diseases and conditions [35].

Clinical assessments were performed at fixed follow-up visits on the first day of each evaluation period, baseline, week 1, week 5, and week 9, while participants continued the assigned mouthrinse protocol during the intervals between visits.

### 4.2. Participants and Ethical Considerations

A total of 175 young adults aged 20–45 years were recruited from the Clinic of “Vasile Goldiș” Western University in Arad, Romania. All participants were systemically healthy and met the predefined inclusion and exclusion criteria. Subjects were randomly allocated into five study groups (three herbal mouthwash groups, one placebo group, and one chlorhexidine control group), with 35 participants in each group.

Inclusion criteria:

- Age between 20 and 45 years, presenting with plaque-induced gingival changes and without clinical signs of periodontitis, including attachment loss, periodontal pocketing > 3 mm, or radiographic bone loss.
- At least 20 natural teeth and no extensive prosthetic rehabilitation.
- No orthodontic or periodontal treatment in the past 6 months.
- Willingness to use only the assigned mouthwash and follow study instructions.
- Provided written informed consent.

Exclusion criteria:

- Known allergy or hypersensitivity to chamomile, sage, or ginger.
- Use of antibiotics, anti-inflammatories, or antiseptic mouthwashes within the last 4 weeks.
- Chronic medical conditions such as diabetes mellitus, hypertension, cardiovascular disease, autoimmune disorders, or other systemic conditions known to affect gingival health or plaque accumulation.
- Pregnant or lactating women.
- Current orthodontic appliances or extensive prosthetic restorations.
- Active caries or acute oral infections at baseline.

### 4.3. Group Allocation and Mouthwash Preparation

Participants were randomly assigned to one of five study groups (n = 35 per group) according to the type of mouthwash used (Table 8):

The three herbal mouthwashes were freshly prepared under standardized conditions by steeping 20 g of dried plant material in 1 L of distilled water for 15 min, followed by filtration and cooling to room temperature. The resulting infusions were stored in sterile amber bottles at 4 °C and used within seven days to ensure stability and microbiological safety. On the first day of the week, patients’ mouthwash supplies were replenished for the entire week.

The placebo mouthwash consisted of distilled water supplemented with a food-grade flavoring agent (mint), glycerol (1%), prepared and stored under identical conditions, ensuring similar appearance and handling without active pharmacological components. The formulation was designed to mimic the sensory characteristics (taste and mouthfeel) of the active mouthwashes while lacking any known antibacterial or anti-inflammatory activity. The chlorhexidine mouthwash was used as a positive control and consisted of a commercially available 0.12% chlorhexidine digluconate solution.

This was a double-blind randomized controlled clinical trial. All mouthwash formulations (herbal, placebo, and chlorhexidine) were dispensed in identical, opaque, and unlabeled containers to ensure indistinguishable appearance.

### 4.4. Rinsing Protocol

Participants were instructed to use the assigned mouthwash three times daily—after breakfast, lunch, and dinner—for twelve consecutive weeks. Each rinsing session consisted of 10 mL of mouthwash for 30 s, followed by abstaining from eating or drinking for 30 min. This standardized dosing approach may represent a deviation from conventional chlorhexidine regimens and could have influenced the magnitude of the observed clinical response in the control group. Therefore, the chlorhexidine-related outcomes should be interpreted in the context of protocol standardization rather than direct equivalence to routine clinical use [36]. To avoid possible inactivation of chlorhexidine by anionic toothpaste surfactants, particularly sodium lauryl sulfate, participants were instructed to rinse no earlier than 30 min after toothbrushing [37].

Participants were also asked to maintain their regular toothbrushing routine (twice daily using a neutral fluoride-free toothpaste) and to refrain from using any other mouthwashes or adjunctive hygiene products during the study period.

### 4.5. Phytochemical Characterization of Herbal Extracts

#### 4.5.1. Determination of Total Phenolic and Flavonoid Content

To obtain preliminary information on the composition of the aqueous extracts, they were analysed by spectral methods, quantifying total phenolic acids and total flavonoid content. For each determination, a Techcomp UV2500 spectrophotometer (Techcomp Ltd., Hong Kong, China) was employed. All determinations were made in triplicate, and the average value was calculated. The results are given as mean ± standard deviation.

**Figure 5:**
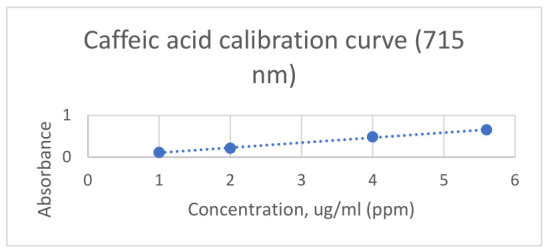
Calibration curve of caffeic acid measured at 715 nm. Standard solutions in the concentration range of 1–5.5 µg/mL were prepared and analyzed spectrophotometrically. Absorbance values showed a linear relationship with concentration, confirming the suitability of the method for quantitative determination of phenolic compounds. The calibration curve was constructed using linear regression analysis (R^2^ ≥ 0.99).

For the determination of total phenolic acid content, 1 mL of each extract was added to 1 mL of phosphotungstic reagent, and the mixture was diluted to 25 mL with 15% sodium carbonate solution. The spectral determination was performed after 2 min of repose at 715 nm against a blank prepared from 1 mL of extract diluted to 25 mL with 15% sodium carbonate solution, according to the Romanian Pharmacopoeia (1993) [38]. For quantification purposes, a calibration curve was built using standard caffeic acid solution (1–5.6 mg/mL). The curve equation is Absorbance = 0.1201 × concentration − 0.0129, and the correlation factor is R^2^ = 0.9971. Figure 5 shows the calibration curve.

**Figure 6:**
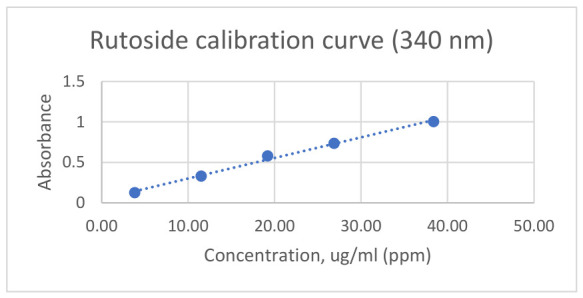
Calibration curve of rutin (rutoside) at 340 nm. Standard solutions ranging from 5 to 40 µg/mL were analyzed spectrophotometrically. A linear relationship between absorbance and concentration was observed, demonstrating the method’s suitability for quantitative determination of flavonoid compounds. The calibration curve was generated using linear regression analysis (R^2^ ≥ 0.99).

The total flavonoid content is determined by adding 1 mL from each extract to 3 mL of 2% aluminium chloride solution and 5 mL of 10% sodium acetate solution. The mixture was brought to 25 mL with methanol. The determinations were carried out after 30 min of incubation period at 340 nm against a solution obtained from 1 mL extract, 8 mL purified water, and brought to 25 mL with methanol, according to the Romanian Pharmacopoeia [38]. For quantification purposes, a calibration curve was constructed using rutoside (rutin) as the reference standard (3.84–38.40 mg/mL). The curve has the following equation: Absorbance = 0.0255 × concentration + 0.0459. Additionally, the correlation factor was R^2^ = 0.9938. In Figure 6, the calibration curve is shown.

#### 4.5.2. LC–MS/MS Analysis of Individual Polyphenolic Compounds

The next step in the analysis will evaluate the individual polyphenols and their content in the extracts studied using LC/MS (Table 9).

For LC/MS analysis, a Shimadzu Nexera I LC/MS-8045 (Kyoto, Japan) UHPLC system equipped with a quaternary pump and autosampler, an ESI probe, and a quadrupole mass spectrometer was used. The separation was carried out on a Luna C18 reversed-phase column (150 mm × 4.6 mm × 3 mm, 100 Å) from Phenomenex (Torrance, CA, USA). The column was maintained at 40 C degrees during the analysis. The mobile phase (see table below) was a gradient of methanol (Merck, Darmstadt, Germany) and ultrapurified water prepared by a Simplicity Ultra Pure Water Purification System (Merck Millipore, Billerica, MA, USA), with formic acid (Merck, Darmstadt, Germany). The methanol and the formic acid were of LC/MS grade. The used flow rate was 0.5 mL/min. The total run time for each chromatographic analysis was 36 min.

**Table 9:** Mobile phase gradient program used for UHPLC–ESI–MS/MS analysis.

Time, min	Methanol (A)	0.1 % Formic Acid in Water (C)
0.00	5	95
3.00	25	75
6.00	25	75
9.00	37	63
13.00	37	63
18.00	54	46
22.00	54	46
26.00	95	5
29.00	95	5
30.00	5	95
36.00	5	95

The detection was performed on a quadrupole rod mass spectrometer operated in electrospray ionisation (ESI) mode, in both negative and positive MRM (multiple reaction monitoring) ion modes (see the table of standards). The interface temperature was set to 300 °C. For vaporisation and as a drying gas, nitrogen was used at 35 psi and 10 L/min, respectively. The capillary potential was set at +3000 V [19,39].

For identification and quantification purposes, several 51 polyphenol standards belonging to phenolic acids, flavonoids and isoflavones classes. Only the standards identified in the studied extracts are included. For each standard, a calibration curve was constructed. The calibration curves, equations and data are included in Table 10 and Table 11. All determinations were made in triplicate, and the results are expressed as mean ± standard deviation.

### 4.6. Examiner Calibration and Data Collection

Two examiners were trained and calibrated before data collection. Calibration was performed by repeated clinical assessment of 10 participants under standardized conditions, with re-evaluation after 7 days; the intra-examiner agreement was κ = 0.88, while the inter-examiner agreement was κ = 0.86.

Clinical examination was performed under standardized clinical conditions using a dental mirror and a WHO periodontal probe. Plaque accumulation was assessed using the Silness–Löe Plaque Index (PI), recorded on four surfaces of each tooth (mesial, distal, buccal, and lingual/palatal), and mean values were calculated for each participant. Gingival clinical status was evaluated using the modified Löe–Silness Gingival Index (Lobene modification), based on the assessment of color, edema, and visual signs of gingival inflammation around the gingival margin [40]. No adverse effects, including tooth discoloration, mucosal irritation, or taste alteration, were reported by participants during the study period. Data were recorded on individual case report forms and transferred to an electronic database using double-entry verification to minimize transcription errors.

### 4.7. Statistical Analysis

Descriptive statistics were used to summarize demographic and clinical variables. Statistical analyses were conducted using Jamovi (v2.5.5). Descriptive statistics were reported as mean ± standard deviation (SD). Differences between groups and time points were analyzed using repeated-measures ANOVA (group × time interaction) with Bonferroni correction for multiple comparisons. Normality was tested using the Shapiro–Wilk test. Statistical significance was set at *p* < 0.05.

### 4.8. Sample Size and Power Analysis

**Figure 7:**
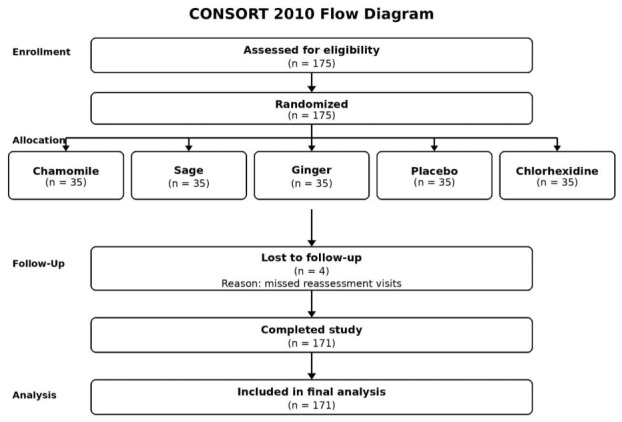
CONSORT flow diagram showing participant enrollment, randomization, follow-up, and analysis.

The required sample size was estimated a priori using GxPower software (version 3.1.9.4). The calculation was based on a repeated-measures ANOVA with a within–between interaction design, including five groups and four repeated measurements. Assuming a small-to-moderate effect size (Cohen’s f = 0.15), an alpha level of 0.05, a desired power of 0.95, a correlation among repeated measures of 0.5, and a nonsphericity correction ε = 1, the minimum total sample size required was 150 participants. To ensure adequate statistical power and allow for potential dropouts, 175 participants were enrolled in the study. During the 12-week follow-up period, 4 participants were lost to follow-up due to missed scheduled reassessment visits, resulting in a final sample of 171 participants who completed all study visits and were included in the repeated-measures analyses. The flow of participants through the study stages, including enrollment, allocation, follow-up, and analysis, is illustrated in Figure 7.

## 5. Conclusions

The present randomized clinical trial provides preliminary evidence that short-term use of chamomile-, sage-, and ginger-based mouthwashes is associated with improvement in clinical signs of plaque-induced gingival inflammation among young adults. All groups improved, but no statistically significant differences were found between the formulations. The rinses were generally well tolerated, and no adverse events were reported during the twelve-week intervention period.

These findings suggest that natural mouthwashes could be safely incorporated as adjuncts to routine oral hygiene, but it remains unclear if they are as effective as conventional antiseptics. Future studies should focus on longer-term randomized controlled trials involving larger and more diverse patient populations, including older adults and patients with systemic comorbidities. Additionally, standardized phytochemical profiling, microbiological evaluation, and host-response biomarker analysis are strongly recommended to better clarify the mechanisms of action and to define their potential role in preventive and therapeutic periodontal care.

## Figures and Tables

**Table 1:** Total Phenolic Acid Content (Caffeic Acid Equivalents) and Total Flavonoid Content (Rutoside Equivalents) of the Investigated Herbal Extracts.

	Chamomille	Sage	Ginger
Total phenolic acid content. expressed in caffeic acid. mg/mL	157.85 ± 1.281	233.68 ± 9.481	32.38 ± 1.850
Total flavonoid content. expressed in rutoside. mg/mL	164.90 ± 2.343	291.50 ± 1.422	21.99 ± 1.101

**Table 2:** Quantitative LC–MS/MS Determination of Individual Polyphenolic Compounds in Chamomile, Sage, and Ginger Extracts (µg/mL).

Polyphenol	Content in Chamomille Extract. µg/mL	Content in Sage Extract. µg/mL	Content in Ginger Extract. µg/mL
Chlorogenic acid	285.50 ± 10.713		
*neo*-Chlorogenic acid	34.60 ± 0.972		
*crypto*-Chlorogenic acid	90.61 ± 3.117		
Caffeic acid		59.24 ± 1.741	
Ferrulic acid	62.95 ± 2.141	17.68 ± 0.995	
Rosmarinic acid		744.27 ± 10.457	
Acacetin	0.46 ± 0.003	0.46 ± 0.003	0.49 ± 0.009
Apigenin	1.32 ± 0.097		
Diosmin	6.29 ± 0.128	4.76 ± 0.171	
Genistin	583.40 ± 10.775	117.68 ± 3.456	
Hyperoside	1.62 ± 0.097		
Luteolin-*7-O*-glucoside	12.17 ± 0.851	13.39 ± 0.771	
Luteolin-*7*-rutinoside		2.65 ± 0.091	
Quercetin-3-glucoside	0.78 ± 0.007		
Tilianin	4854.36 ± 25.165	6501.78 ± 88.311	26.12 ± 0.971

**Table 3:** Baseline demographic and oral hygiene characteristics of participants (N = 175).

Variable	Chamomile (n = 35)	Sage (n = 35)	Ginger (n = 35)	Placebo (n = 35)	Chlorhexidine (n = 35)
Age (years), mean ± SD	27.51 ± 5.97	27.00 ± 5.91	27.86 ± 5.87	31.20 ± 6.01	31.17 ± 6.37
Gender (Male/Female), n/n	14/21	13/22	16/19	13/22	18/17
Residence (Urban/Rural), n/n	27/8	30/5	25/10	30/5	27/8
Toothbrushing frequency (times/day), mean ± SD	2.09 ± 0.61	2.26 ± 0.51	2.14 ± 0.60	2.43 ± 0.66	2.51 ± 0.61
Mouthwash use frequency (times/day), mean ± SD	2.77 ± 1.03	2.37 ± 0.97	2.29 ± 0.96	2.06 ± 1.47	2.03 ± 1.65

**Table 4:** Baseline plaque and gingival indices across study groups (N = 175).

Variable	Chamomile (n = 35)	Sage (n = 35)	Ginger (n = 35)	Placebo (n = 35)	Chlorhexidine (n = 35)	*p*-Value
Silness–Löe Plaque Index (T0), mean ± SD	1.57 ± 0.56	1.37 ± 0.77	1.40 ± 0.74	1.41 ± 0.37	1.39 ± 0.38	>0.05
Modified Löe and Silness Gingival Index (Lobene, T0), mean ± SD	0.29 ± 0.46	0.23 ± 0.43	0.34 ± 0.48	0.30 ± 0.14	0.26 ± 0.15	>0.05

**Table 5:** Summary of repeated measures ANOVA results for the Silness–Löe Plaque Index.

Effect	SS	df	MS	F	*p*	η^2^_p_	Interpretation
Within-subjects effects							
Time	115.90	2.18, 370.07	38.63	214.60	<0.001	0.56	Significant effect of time—plaque accumulation decreased markedly over the study period
Time × Type of mouthwash used	89.00	8.72, 370.07	7.42	41.20	<0.001	0.49	Significant interaction—plaque reduction over time differed substantially between mouthwash types
Residual (within)	91.80	510	0.18	—	—	—	—
Between-subjects effects							
Type of mouthwash used	57.10	4	14.27	38.00	<0.001	0.47	Significant differences in plaque levels between mouthwash groups
Residual (between)	63.90	170	0.38	—	—	—	—

Residual df = 510 reflects listwise exclusion of participants with incomplete follow-up data (n = 171 valid cases). Greenhouse–Geisser correction applied for within-subject effects.

**Table 6:** Summary of repeated measures ANOVA results for the Gingival Index.

Effect	SS	df	MS	F	*p*	η^2^_p_	Interpretation
Within-Subjects effects							
Time	34.60	2.42, 410.60	11.54	47.29	<0.001	0.22	Significant effect of time—gingival index scores changed significantly over the study period
Time × Type of mouthwash used	25.90	9.68, 410.60	2.16	8.84	<0.001	0.17	Significant interaction—gingival response over time differed between mouthwash types
Residual (within)	124.40	510	0.24	—	—	—	—
Between-subjects effects							
Type of mouthwash used	8.96	4	2.24	10.00	<0.001	0.19	Significant differences in gingival index between mouthwash groups
Residual (between)	38.02	170	0.22	—	—	—	—

**Table 7:** The comparable clinical effectiveness in reducing dental plaque and gingival index scores of all three herbal mouthwashes.

Index	F (df)	*p*-Value	η^2^_p_	Significant Time Effect	Time × Group Interaction	Between-Group Effect	Interpretation
Silness–Löe Plaque Index	F (2.18, 370.07) = 214.6	<0.001	0.56	✔ Yes	✔ Yes (*p* < 0.001)	✔ Yes (*p* < 0.001)	Strong and sustained reduction in plaque accumulation over time, with significantly different response patterns between mouthwash types
Modified Löe & Silness Gingival Index (Lobene)	F (2.42, 410.60) = 47.29	<0.001	0.22	✔ Yes	✔ Yes (*p* < 0.001)	✔ Yes (*p* < 0.001)	Significant improvement in gingival index scores over time, with moderate but meaningful differences between treatment groups

**Table 8:** Composition of the Mouthwash Formulations.

Group	Mouthwash Type	Main Active Components	Concentration
G1	*Matricaria chamomilla* (Chamomile)	Apigenin, bisabolol	2% aqueous infusion
G2	*Salvia officinalis* (Sage)	Rosmarinic acid, cineole	2% aqueous infusion
G3	*Zingiber officinale* (Ginger)	Gingerols, shogaols	2% aqueous infusion
G4	Placebo	Distilled water, mint flavour, glycerol	
G5	Chlorhexidine	Chlorhexidine digluconate	0.12% solution

**Table 10:** Calibration Parameters and Validation Data for the LC–MS/MS Quantification of Polyphenolic Standards.

Name of Standard	Origin	Concentration Range, mg/mL	Calibration Curve Equation	Correlation Factor	Detection Limit, mg/mL	Quantification Limit, mg/mL
Caffeic acid	Phytolab, Vestenbergsgreuth, Germany	11.300–113.000	Area = 45,631.7 × conc [mg/mL] + 507,513	0.9908	22.24	44.49
Chlorogenic acid	Phytolab, Vestenbergsgreuth, Germany	14.000–140.000	Area = 397,772 × conc [mg/mL] + 3,567,660	0.9954	17.95	35.90
Cryptochlorogenic acid	Merck, Darmstadt, Germany	10.000–100.000	Area = 97,960.4 × conc [mg/mL] + 418,649	0.9989	8.55	17.09
Neochlorogenic acid	Merck, Darmstadt, Germany	10.000–100.000	Area = 208,756 × conc [mg/mL] + 81,773.1	0.9988	0.78	1.57
Ferulic acid	Phytolab, Vestenbergsgreuth, Germany	10.400–104.000	Area = 13,138 × conc [mg/mL] – 67,118.4	0.9760	20.43	30.65
Rosmarinic acid	Phytolab, Vestenbergsgreuth, Germany	11.100–111.000	Area = 151,146 × conc [mg/mL] + 1.08 × 10^6^	0.9924	14.29	28.58
*Trans-para*- coumaric acid	Phytolab, Vestenbergsgreuth, Germany	10.200–102.000	Area = 262,008 × conc [mg/mL] + 3.07 × 10^6^	0.9929	23.43	46.87
Acacetin	Phytolab, Vestenbergsgreuth, Germany	1.200–12.000	Area = 2.02 × 10^6^ × conc [mg/mL] – 535,751	0.9532	1.06	1.59
Genistin	Fluka, Sigma-Aldrich, USA	1.790–17.900	Area = 56,209.1 × conc [mg/mL] + 123,966	0.9859	4.41	8.82
Hyperoside	Phytolab, Vestenbergsgreuth, Germany	1.070–10.700	Area = 4.61 × 10^6^ × conc [mg/mL] + 2.91 × 10^6^	0.9961	1.26	2.52
Luteolin-*7-O*-glucoside	Phytolab, Vestenbergsgreuth, Germany	0.285–2.850	Area = 2.51 × 10^6^ × conc [mg/mL] + 377,486	0.9856	0.30	0.60
Rutoside	Phytolab, Vestenbergsgreuth, Germany	0.960–9.600	Area = 6.51 × 10^6^ × conc [mg/mL] + 4.55 × 10^6^	0.9557	1.40	2.80
Tilianin	Phytolab, Vestenbergsgreuth, Germany	3.100–31.000	Area = 5880.86 × conc [mg/mL] + 28,495.1	0.9901	9.69	19.38
Apigenine	Phytolab, Vestenbergsgreuth, Germany	0.105–1.050	Area = 7.73 × 10^6^ × conc [mg/mL] + 500,105	0.9852	0.13	0.26
Quercetine-3-glucoside	Phytolab, Vestenbergsgreuth, Germany	1.200–12.000	Area = 1.08 × 10^6^ × conc [mg/mL] + 646,984	0.9869	1.20	2.39
Diosmin	Phytolab, Vestenbergsgreuth, Germany	1.400–14.000	Area = 1.08 × 10^6^ × conc [mg/mL] + 124,993	0.9997	0.24	0.47
Luteolin-7-rutinoside	Phytolab, Vestenbergsgreuth, Germany	5.700–57.000	Area = 1.29 × 10^6^ × conc [mg/mL] + 6.70 × 10^6^	0.9886	10.39	20.77

**Table 11:** MRM Transitions and Mass Spectrometric Parameters for LC–MS/MS Identification of Polyphenolic Compounds.

Name of Standard	*m*/*z*. and Main Transition	MRM	Other Transitions
Caffeic acid	179.0 > 135.0	Negative	179.0 > 134.0 179.0 > 89.0
Chlorogenic acid	353.05 > 191.0	Negative	353.05 > 127.0 353.05 > 93.0 353.05 > 85.0
Cryptochlorogenic acid	353.15 > 134.95	Negative	353.15 > 191.0 353.15 > 173.0 353.15 > 93.0 353.15 > 85.0
Neochlorogenic acid	353.0 > 135.0	Negative	353.0 > 191.0 353.0 > 85.0
Ferulic acid	193.05 > 133.1	Negative	193.05 > 178.0
Rosmarinic acid	359.0 > 161.0	Negative	359.0 > 133.0
*Trans-para* coumaric acid	163.0 > 119.05	Negative	163.0 > 117.0 163.0 > 93.0
Acacetin	283.1 > 268.0	Negative	
Genistin	431.0 > 268.0	Negative	431.0 > 240.0
Hyperoside	463.1 > 300.1	Negative	463.1 > 301.0
Luteolin-*7-O*-glucoside	447.0 > 284.9	Negative	
Rutoside	609.0 > 300.0	Negative	609.0 > 301.0 609.0 > 271.0
Tilianin	447.1 > 285.0	Negative	
Apigenine	269.0 > 151.0	Negative	269.0 > 121.0 269.0 > 118.0 269.0 > 117.0 269.0 > 107.0
Quercetine-3-glucoside	463.0 > 301.0	Negative	
Diosmin	607.2 > 299.0	Negative	607.2 > 341.0 607.2 > 284.0 607.2 > 265.0 607.2 > 150.0
Luteolin-7-rutinoside	393.15 > 285.0	Negative	393.15 > 297.0

## Data Availability

All data regarding this manuscript can be requested from the corresponding author.
